# High-Molecular-Weight Hyaluronic Acid Vehicle Can Deliver Gadolinium Into the Cochlea at a Higher Concentration for a Longer Duration: A 9.4-T Magnetic Resonance Imaging Study

**DOI:** 10.3389/fneur.2021.650884

**Published:** 2021-06-24

**Authors:** Yu-Jung Hwang, Mina Park, Moo Kyun Park, Jun Ho Lee, Seung Ha Oh, Myung-Whan Suh

**Affiliations:** ^1^Department of Otorhinolaryngology-Head and Neck Surgery, Seoul National University Hospital, Seoul, South Korea; ^2^Interdisciplinary Program in Neuroscience, College of Natural Sciences, Seoul National University, Seoul, South Korea; ^3^Department of Otorhinolaryngology-Head and Neck Surgery, Seoul Medical Center, Seoul, South Korea

**Keywords:** magnetic resonance imaging, hyaluronic acid, gadolinium, drug delivery, cochlea, intratympanic injection

## Abstract

Intratympanic (IT) gadolinium (Gd) injection is one method of delivering Gd into the inner ear to evaluate the amount of endolymphatic hydrops (EH) using magnetic resonance imaging (MRI). As Gd is usually prepared in a fluid form mixed with saline, Gd injected into the middle ear drains easily through the Eustachian tube within several hours. High-molecular-weight (hMW) hyaluronic acid (HA) is an ideal vehicle for IT Gd due to its viscous and adhesive properties. The present study was performed to elucidate whether novel hMW HA is superior to conventional HA in delivering Gd into the inner ear in the short term. The second aim was to verify the long-term Gd delivery efficiency of hMW HA compared to the standard-of-care vehicle (saline). IT Gd injection and 3D T1-weighted MRI were performed in 13 rats. For the short-term study (imaging after 1, 2, and 3 h), the left ear was treated with hMW HA+Gd and the right ear with conventional HA+Gd. For the long-term study (imaging after 1, 2, 3, and 4 h, 1 – 3 days, and 7 – 10 days), the left ear was treated with hMW HA+Gd and the right ear with saline+Gd. Signal intensities (SIs) in the scala tympani (ST) and scala vestibuli (SV) were quantified. Compared to conventional HA, signal enhancement was 2.3 – 2.4 times greater in the apical and middle turns after hMW HA+Gd injection (SV at 1 h). In comparison to the standard-of-care procedure, the SI was not only greater in the short term but the higher SI also lasted for a longer duration. On days 7 – 10 after IT Gd delivery, the SI in the basal turn was 1.9 – 2.1 times greater in hMW HA+Gd-treated ears than in saline IT Gd-treated ears. Overall, hMW HA may be a useful vehicle for more efficient IT Gd delivery. Gd enhancement in the cochlea improved approximately two-fold when hMW HA was used. In addition, this greater enhancement lasted for up to 7 – 10 days. Repeated MRI of EH may be possible for several days with a single IT hMW HA+Gd delivery.

## Introduction

Intratympanic (IT) gadolinium (Gd) injection is one method of delivering Gd into the inner ear to evaluate the amount of endolymphatic hydrops (EH). Classically, EH has been considered a pathognomonic sign of Ménière's disease (MD) (1). However, until recently, the detection of EH in a live MD patient was not possible, and histological evaluation could only be performed postmortem. With advances in magnetic resonance imaging (MRI) technology, it is now possible to separately image the perilymphatic and endolymphatic spaces (2–4). This new imaging technique may allow objective detection of the presence of EH (5, 6) and characterization of the physiology of intracochlear fluid dynamics in living humans.

For better MRI outcomes, a more efficient and long-lasting Gd delivery method is needed. The duration of Gd enhancement by conventional IT methods is limited to 1 or 2 days because Gd is usually injected in a fluid form mixed with saline (5, 7), and fluidic Gd injected into the middle ear drains easily through the Eustachian tube within several hours. The best time point to image the inner ear with the maximum Gd enhancement effect is ~24 h after IT Gd injection (8, 9). This problem applies to not only Gd but also other therapeutic drugs, such as dexamethasone, delivered via the IT route. The dexamethasone levels in guinea pig perilymph peaked at 1 h after IT injection and decreased quickly to ~1% of the initial concentration by 12 h (10). Vehicles that can last for several days in the middle ear and elute drug in a sustained matter would compensate for this disadvantage of IT delivery (11). If the drug/vehicle does not drain through the Eustachian tube, it will have a higher probability of coming into contact with the round and oval windows, thus facilitating diffusion of the drug into the perilymphatic space consisting of the scala tympani (ST) and scala vestibule (SV). That is, Gd delivered via an effective vehicle will result in stronger enhancement of MRI signals for a longer duration in the ST and SV (12).

In the present study, we focused on high-molecular-weight (hMW) hyaluronic acid (HA) as a potential vehicle. HA is an anionic, non-sulfated glycosaminoglycan that is biodegradable (13). It is used in the regeneration of various tissues, such as vocal folds, cartilage, and salivary glands. It has also been studied as a carrier in targeted cell delivery, tumor-targeting drug delivery, and the sustained release of drugs (14, 15). The vehicle used in this study is novel in that it had a very high molecular weight (16). Whereas, conventional HA usually has a molecular weight of 1,000–3,000 kDa, this novel HA preparation has a molecular weight of 5,000–10,000 kDa. The high molecular weight results in an extremely viscous HA (viscoelasticity of 100–200 Pa·s at 1 Hz) with good adhesive properties. In other parts of the body, previous studies have shown superior Gd enhancement using hMW HA as the drug delivery vehicle (17).

In this study, we verified the utility of hMW HA as an IT Gd vehicle in the ear. We hypothesized that hMW HA would last for a long time in the middle ear due to its viscosity and adhesive properties, which may enable a higher efficiency of Gd delivery, i.e., a higher concentration of Gd in the inner ear and longer duration of Gd delivery compared to the standard-of-care procedure (IT Gd delivery *via* saline). The present study was performed to elucidate whether the novel hMW HA is superior to conventional HA in delivering Gd into the inner ear in the short term and to compare the long-term Gd delivery efficiency of hMW HA with that of the standard-of-care vehicle.

## Materials and Methods

All experiments were approved by the Institutional Animal Care and Use Committee of Seoul National University Hospital (no. 18-0046-C2A0), and animals were maintained in a facility accredited by the Association for Assessment and Accreditation of Laboratory Animal Care International (#001169) in accordance with the National Research Council's Guide for the Care and Use of Laboratory Animals, 8th edition (2010). A total of 12 Sprague–Dawley rats (weight = 200–250 g) with normal hearing were used in the study. The vehicle used to deliver Gd was different between the left and right ears within the same animals, whereas the Gd was identical. This allowed us to verify the effects of different vehicles on MRI enhancement independent of differences in Gd. In the first study, the vehicle was hMW HA in the left ear and conventional HA in the right ear. In the second study, the vehicle was hMW HA in the left ear and saline in the right ear.

### Drugs and Vehicles

Different Gd preparations (drugs) were used for the first and second studies: Gd-diethylenetriamine pentaacetic acid (DTPA; Magnevist®, Bayer Schering Pharma AG, Berlin, Germany) was used for the first study and Gd-DO3A-butrol (Gadovist®, Bayer Schering Pharma AG) was used for the second study. These Gd preparations were selected based on the goal of the study and the inner ear enhancement properties as summarized in Table 1 (18, 19). Gd-DTPA was selected for the first study (short-term comparison between two HA vehicles) because it has been reported to be superior in enhancing the whole cochlea (from apex to base) in the short term (4). Gd-DO3A-butrol was selected for the second study (long-term comparison between HA and standard-of-care vehicle) because it was reported to exhibit the strongest enhancement (in the basal turn) over a longer duration (3). The Gd preparations were diluted to a final concentration of 0.5 mol/L in the first and second studies.

**Table 1:** Properties of the two gadolinium contrast agents used in this study.

	**Gd-DTPA**	**Gd-DO3A-butrol**
**Chemical properties**		
Molecular structure	Linear, ionic	Macrocyclic, non-ionic
Concentration (mol/L)	0.5	1.0
Thermodynamic stability constant (log Keq)	22.1	21.8
Conditional stability constant at pH 7.4	18.1	14.7
Osmolality (Osm/kg)	1.96	1.6
Viscosity (mPa·s at 37°C)	2.9	5.0
T1 relaxivity (L/mmol·s^−1^) 1.5 T, plasma	4.1	5.2
T1 relaxivity (L/mmol·s^−1^) 3 T, plasma	3.7	5.0
Metal chelate (mg/ml)	469	605
Excess chelate (mg/ml)	0.4	0.5
**Signal intensity in the cochlea after intratympanic Gd delivery**		
Apical turn, SV (after 1 and 4 h)	1.71 and 2.22	0.98 and 1.74
Middle turn, SV (after 1 and 4 h)	2.60 and 2.21	2.02 and 1.95
Basal turn, SV (after 1 and 4 h)	2.02 and 2.18	2.54 and 2.38

*Gd, gadolinium; SV, scala vestibule*.

The specific composition and properties of the vehicles are reported elsewhere (16). Briefly, the hMW HA (MNH Bio Co. Ltd., Hwaseong-si, Republic of Korea) is a non-cross-linked and non-animal-based HA with a molecular weight of 5,000–10,000 kDa and a concentration of 20 mg/ml (Table 2). The conventional HA is also a non-cross-linked and non-animal-based HA. However, the concentration was 10 mg/ml and the molecular weight was 1,000–3,000 kDa. Physiological saline (0.9% sodium chloride in water) was considered the standard-of-care vehicle. The difference in viscosity between the three vehicles can be understood intuitively in Supplementary Video 1. When 20 μl of each vehicle was pipetted onto a vertical glass surface, hMW HA adhered to the glass with no flow, conventional HA flowed down slowly, and saline dripped down instantaneously. The drug and vehicle were physically mixed by stirring.

**Table 2:** Properties of the three vehicles used in this study.

	**hMW HA**	**Conventional HA**	**Saline**
Molecular weight (kDa)	5,000–10,000	1,000–3,000	0.05844
Concentration of HA (mg/ml)	20	10	0
Types of HA	Non-cross-linked HA, Non-animal-based HA	Non-cross-linked HA, Non-animal-based HA	Not applicable

*hMW, high molecular weight; HA, hyaluronic acid*.

### IT Gd Administration

IT injection was performed as previously described (3, 4). The animals were anesthetized with 33 mg/kg zoletil (Virbac, Westlake, TX, USA) and 8 mg/kg xylazine (Bayer AG, Leverkusen, Germany). The tympanic membrane was evaluated using a surgical microscope (Opmi Pico, Zeiss, Oberkochen, Germany). An air hole was made in the anterior inferior quadrant of the tympanic membrane with a 24-gauge intravenous catheter needle (Becton, Dickinson and Company, Franklin Lakes, NJ, USA). An average of 51.4 ± 10.9 μl of drug/vehicle was injected into the middle ear (bulla) through the posterior inferior quadrant of the tympanic membrane.

The quality of drug/vehicle injection was classified according to the following three grades: poor, the middle ear cavity filled with ≤ 10 μl of drug/vehicle; fair, more than half of the middle ear cavity filled; and good, the middle ear cavity completely filled without air bubbles. Overall, we achieved good or fair injections in all animals in this study. The time interval between the first injection in the first ear and the second injection in the other ear was not more than 2 min. After injecting the drug/vehicle, the rats were maintained in a prone position without any tilting until the end of the experiment.

### MRI Measurements and Quantitative Analysis of Signal Intensities

A 9.4-T animal MRI scanner (9.4T/160AS, Agilent Technologies, Santa Clara, CA, USA) was used with an imaging protocol identical to that in our previous studies (3, 4). Initially, scout images were acquired using a 2D gradient echo sequence in all three directions. The imaging parameters were as follows: repetition time (TR)/echo time (TE) = 16.9/2.8 ms, flip angle (FA) = 30°, field of view (FOV) = 40 × 40 mm^2^, matrix size = 128 × 128, seven slices, slice thickness (TH) = 2 mm (no gap), bandwidth = 50 kHz, and an average of two signals. The 3D images were subsequently acquired using 3D fast spin echo with the following imaging parameters: TR/TE = 500/12 ms, FA = 90/180°, echo train length = 8, FOV = 14 × 7 × 7 mm^3^ (slab thickness = 7 mm), matrix size = 128 × 64 × 64, bandwidth = 50 kHz, and an average of six signals. Spatial saturation was performed in all three directions with two saturation bands for each direction. In MRI, both the ST and SV were enhanced, whereas the scala media (SM), an endolymphatic space, was not.

**Figure 1:**
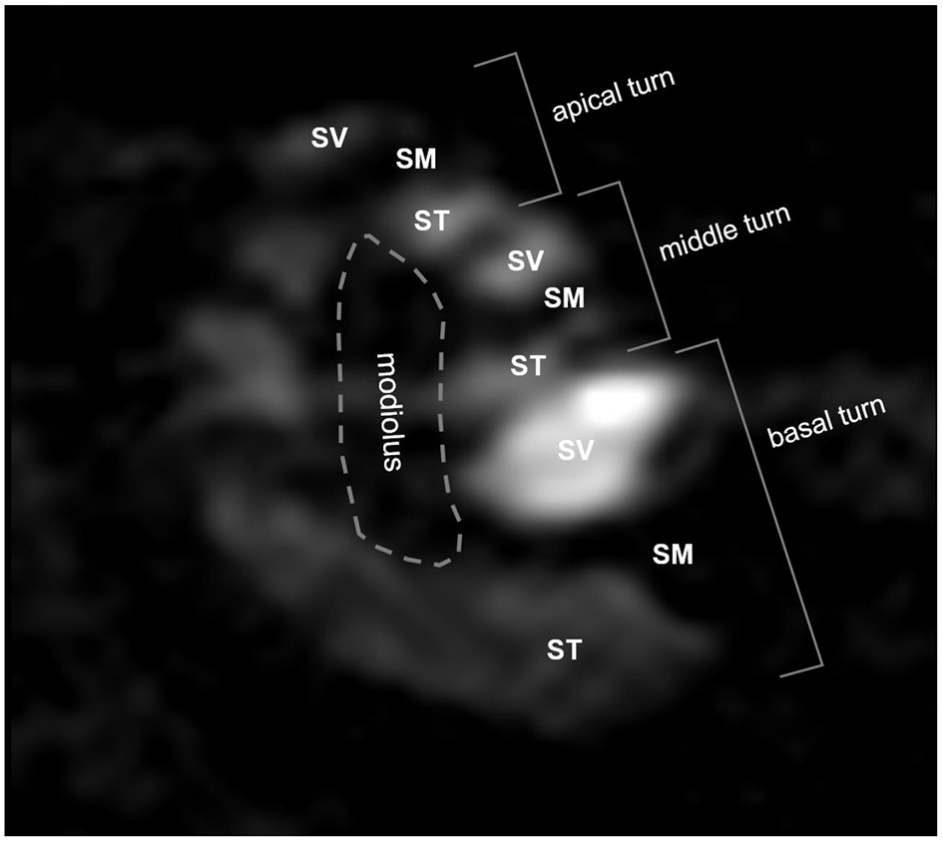
Three-dimensional magnetic resonance imaging of rat cochlea at 2 h after intratympanic (IT) gadolinium (Gd)-diethylenetriamine pentaacetic acid (DTPA) injection. Although the scala tympani (*ST*) and scala vestibuli (*SV*) were enhanced by Gd, the scala media (*SM*) was not. Based on this enhancement pattern, we were able to identify the three compartments (ST, SV, and SM) and the three turns (apical, middle, and basal) of the cochlea. The signal intensity (SI) in each compartment in each turn was quantified for further comparison between the vehicles.

Signal intensities (SIs) were analyzed quantitatively from 2D Digital Imaging and Communications in Medicine (DICOM) files. Due to Gd enhancement, the ST and SV were defined as areas of bright SI in the lower and upper parts of the SM (Figure 1). When it was not possible to segregate the ST and SV due to a delayed enhancement effect, especially in the apical turn, the intensity was considered to be zero. The brainstem in the midline was used as a reference for SI normalization. The normalized SI was defined as: (normalized SI) = (intensity of the compartment of interest)/(intensity of the reference area). The normalized SI was analyzed quantitatively in the SV and ST as described in our previous report (4).

IT Gd injection was only performed once for all ears. However, a repeated series of MRI was performed on the day of IT Gd injection (at 1, 2, and 3 h after IT Gd injection). In the second study, additional imaging was performed several days later (1–3 and 7–10 days) to evaluate the long-term effects.

## Results

### Study 1: Short-Term Differences Between Using hMW HA and Conventional HA

**Figure 2:**
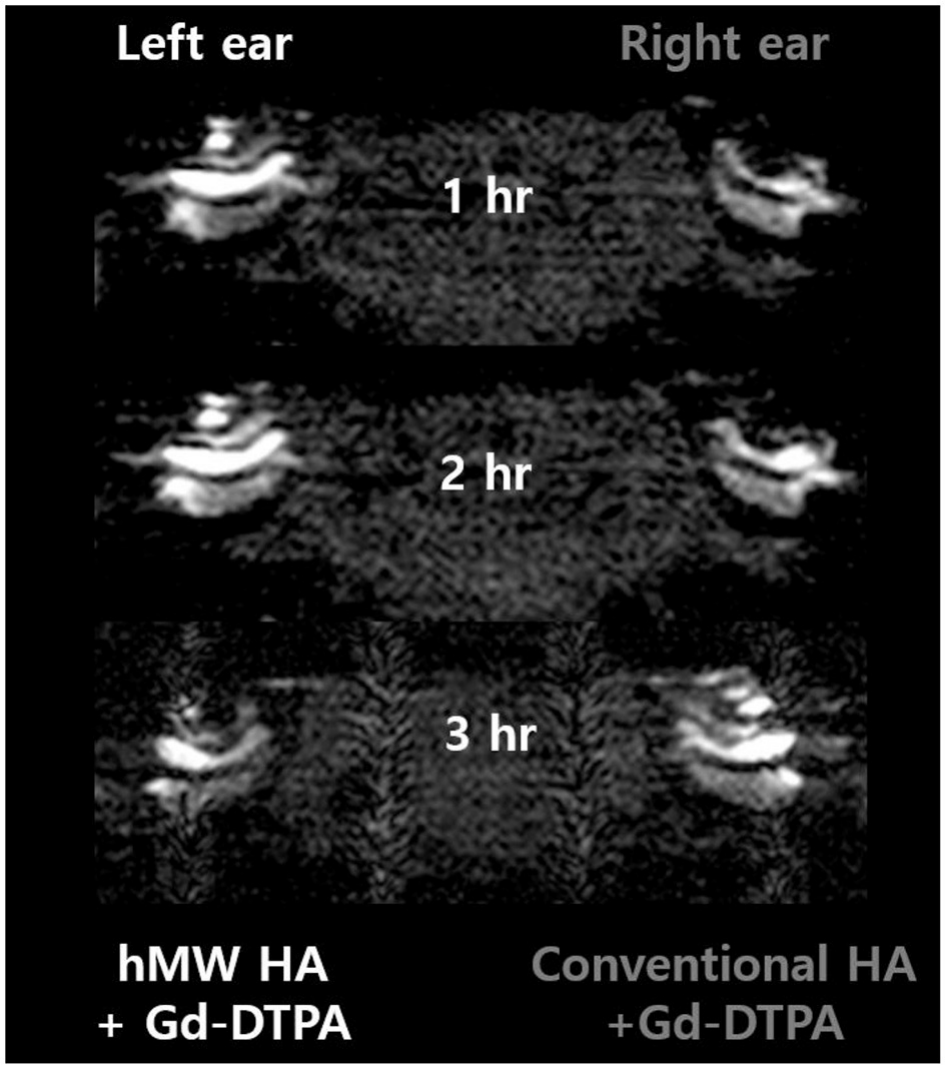
Short-term cochlear enhancement patterns after intratympanic (IT) gadolinium (Gd) injection with two different vehicles. Gd-DTPA was delivered using a high-molecular-weight (hMW) hyaluronic acid (HA) vehicle in the left ear and a conventional HA vehicle in the right ear. Qualitatively, the left cochlea, which was treated with Gd loaded in hMW HA, was brighter than the contralateral cochlea.

**Figure 3:**
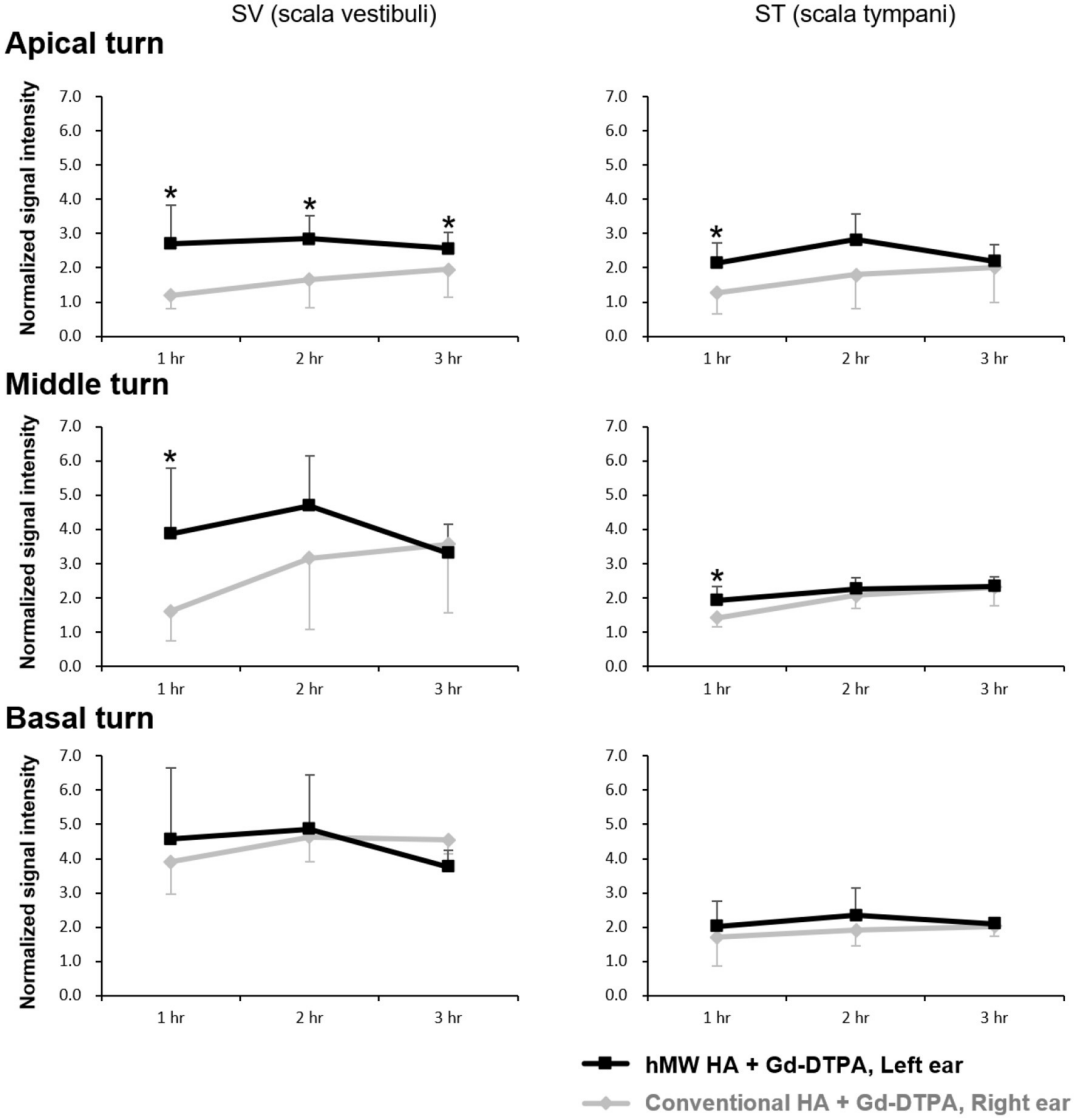
Quantification of short-term normalized signal intensities (SIs) in the scala vestibuli (SV) and scala tympani (ST) after intratympanic (IT) gadolinium (Gd) injection with two different vehicles. The SI was higher when Gd-DTPA was delivered with the high-molecular-weight (hMW) hyaluronic acid (HA) vehicle compared to the conventional HA vehicle. This difference was most significant at 1 h after IT Gd injection in the apical and middle turns. The SI in the basal turn was similar between the two vehicles. *p < 0.05.

Figure 2 shows the representative MRI results of an animal that was injected with hMW HA+Gd in the left ear and conventional HA+Gd in the right ear. The number in the center represents the time elapsed from IT Gd delivery to the time point of MRI. In all animals, the left ear appeared brighter than the right ear. The SI was greater in the ear injected with hMW HA+Gd compared to that in the ear injected with conventional HA+Gd (Figure 3). This difference was most pronounced in the SV at 1 h in the middle turn (normalized SI = 3.9 ± 1.9 vs. 1.6 ± 0.9, respectively, *p* = 0.028) and apical turn (normalized SI = 2.7 ± 1.1 vs. 1.2 ± 0.4, respectively, *p* = 0.027). The difference between the ears decreased over time, but was still statistically significant in the apical turn after 2 h (normalized SI = 2.8 ± 0.7 vs. 1.7 ± 0.8, respectively, *p* = 0.046) and 3 h (normalized SI = 2.6 ± 0.5 vs. 2.0 ± 0.8, respectively, *p* = 0.027). A similar pattern of greater enhancement by hMW HA+Gd was also identified in the ST, especially at 1 h (Figure 3). In the basal turn, the SI was similar between the two groups throughout the whole experimental period; the SI in the SV was similarly high (saturated) and the SI in the ST was similarly low with both vehicles.

### Study 2: Long-Term Differences Between Using hMW HA and Saline

**Figure 4:**
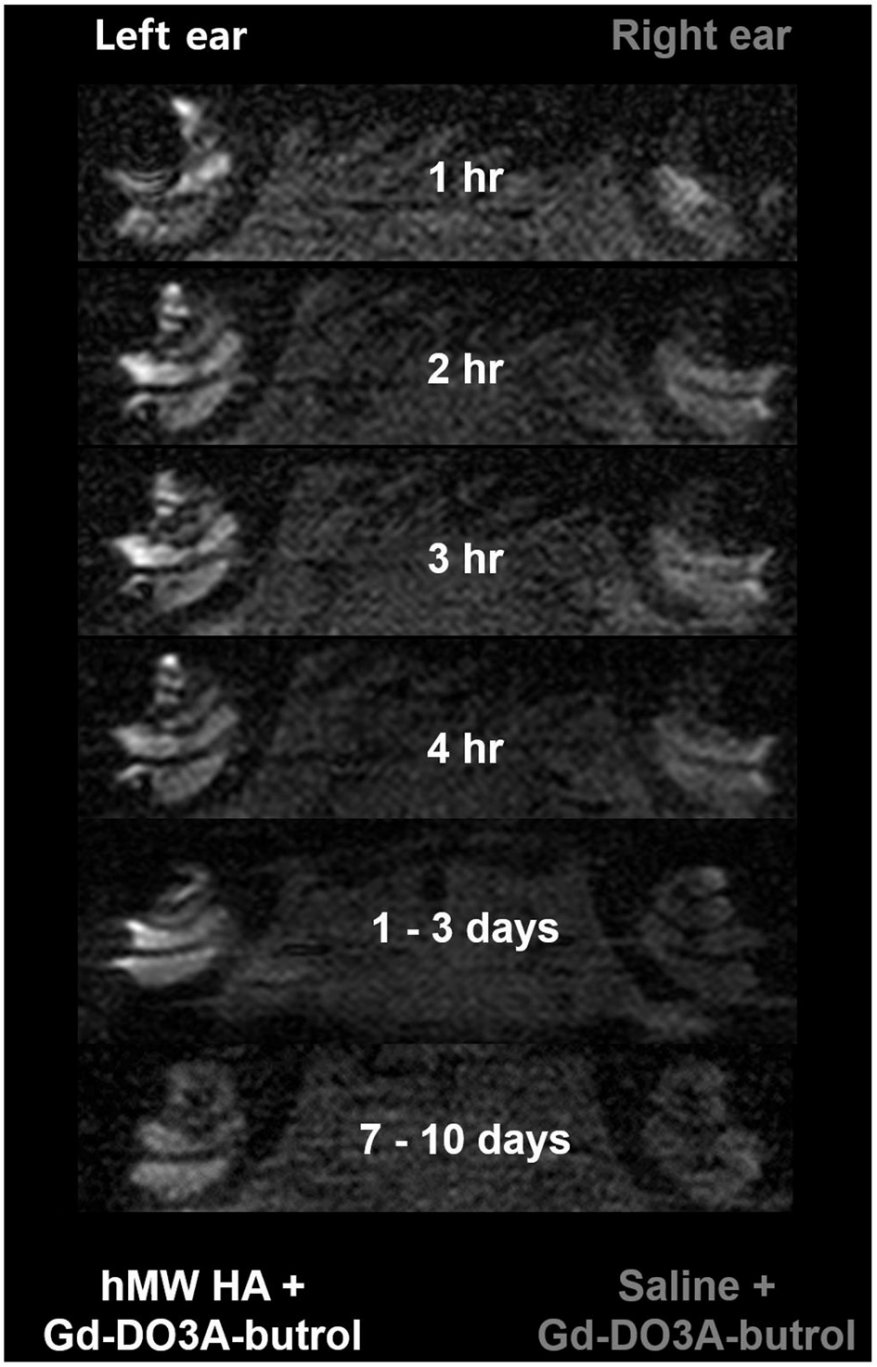
Long-term cochlear enhancement patterns after intratympanic (IT) gadolinium (Gd) injection with two different vehicles. Gd-DO3A-butrol was delivered using a high-molecular-weight (hMW) hyaluronic acid (HA) vehicle in the left ear and saline in the right ear. Qualitatively, the left cochlea, which was treated with Gd loaded in hMW HA, was brighter than the contralateral cochlea.

**Figure 5:**
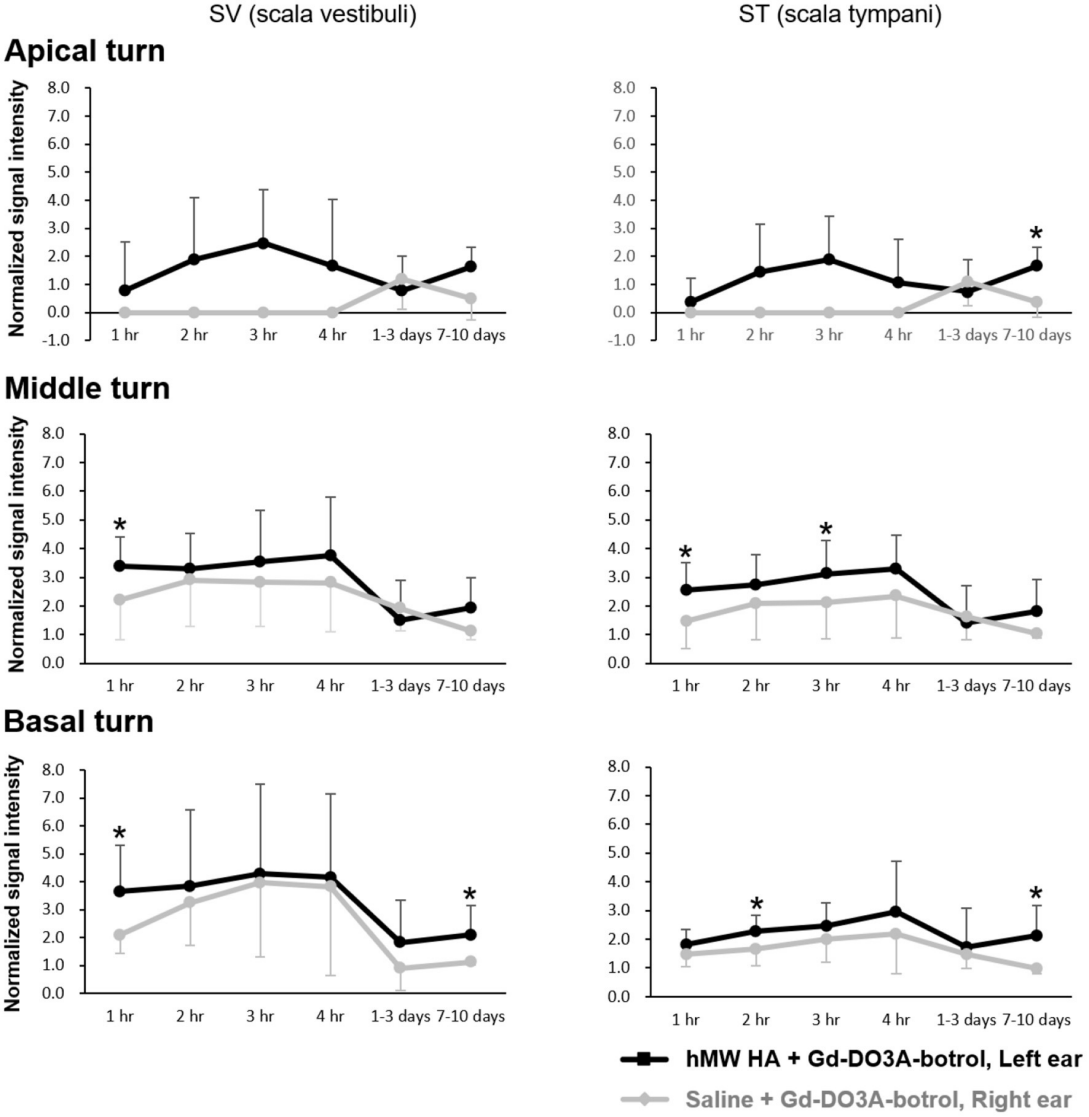
Quantification of long-term normalized signal intensities (SIs) in the scala vestibuli (SV) and scala tympani (ST) after intratympanic (IT) gadolinium (Gd) injection with two different vehicles. The SI was higher when Gd-DO3A-butrol was delivered in a high-molecular-weight (hMW) hyaluronic acid (HA) vehicle instead of saline. This difference was most significant at 1 h after IT Gd injection in the basal and middle turns. The significant elevation in SI lasted for 7–10 days after hMW HA+Gd injection in the basal (SV and ST) and apical (ST) turns. *p < 0.05.

Figure 4 shows the representative MRI results of an animal that was injected with hMW HA+Gd in the left ear and saline+Gd in the right ear. In all animals, the left ear appeared brighter than the right ear. The SI was quantified (Figure 5) and was higher in the ear injected with hMW HA+Gd than in the ear injected with saline+Gd. This difference was most pronounced in the basal and middle turns. That is, the SI was significantly higher in hMW HA+Gd-treated ears during the first 1 h in the SV of the basal turn (normalized SI = 3.6 ± 1.7 vs. 2.1 ± 0.7, respectively, *p* = 0.012) and middle turn (normalized SI = 3.4 ± 1.0 vs. 2.2 ± 1.4, respectively, *p* = 0.035). The SI was also significantly higher after 7–10 days in the basal turn of the SV (normalized SI = 2.1 ± 1.0 vs. 1.1 ± 0.1, respectively, *p* = 0.046) and ST (normalized SI = 2.1 ± 1.0 vs. 1.0 ± 0.2, respectively, *p* = 0.028). The apical turn was only enhanced by hMW HA+Gd injection during the first 4 h. The apical turn in saline+Gd-treated ears was slightly enhanced after 1–3 days. However, after 7–10 days, the apical SI in the ST was significantly lower in saline+Gd-treated ears than that in hMW HA+Gd-treated ears (normalized SI = 1.7 ± 0.7 vs. 0.4 ± 0.5, respectively, *p* = 0.043).

## Discussion

The results of this study showed that hMW HA is potentially applicable as a vehicle for more efficient IT Gd delivery into the cochlea. Compared to conventional HA, the MRI signal enhancement was 2.3–2.4 times greater in the apical and middle turns upon hMW HA+Gd injection (SV at 1 h). Greater enhancement is important for clearly demarcating the endolymphatic space from the perilymphatic space (20). Clinically, clear perilymphatic enhancement is important for identifying EH in subjects with MD with a high sensitivity and specificity (21). In comparison to the standard-of-care procedure (IT Gd delivery via saline), the SI was not only greater in the short term but the higher SI also lasted for a longer duration. On days 7–10 after IT Gd delivery, the SI in the basal turn was 1.9–2.1 times greater in hMW HA+Gd-treated ears than in ears treated with standard-of-care IT Gd. As the maximum SI was 2.2 (basal turn ST at 4 h) with standard-of-care IT Gd, an SI of 2.1 up to 7–10 days after hMW HA treatment seemed to represent a crucial improvement. With a single IT Gd injection, MD patients can be imaged on several different days to evaluate serial changes in the amount of EH. Due to the short-lasting effect of standard-of-care IT Gd, this was not possible previously without repeated IT Gd injections. Repeated IT Gd deliveries are not recommended as these are associated with a risk of middle ear infection (11) and ototoxic inner ear damage (4). Here, we report the superior outcomes (stronger and long-lasting enhancement) of using hMW HA as an IT Gd delivery vehicle.

The greater and longer-lasting MRI signal enhancement with hMW HA use is probably related to the mucoadhesive properties of the vehicle (22). Due to its high viscosity, hMW HA behaves similarly to a thick gel that adheres to the adjacent tissue surface (23). Meanwhile, a liquid formulation of a drug/vehicle injected into the middle ear is assumed to last less than several hours before draining through the Eustachian tube (24). The Eustachian tube is an anatomical structure that connects the middle ear with the nasopharynx and is approximately 35 mm in length and 1.5–2.5 mm in diameter (25). When the tensor veli palatini and levator veli palatini muscles contract (during swallowing or jaw movements), the cartilaginous part of the Eustachian tube opens and facilitates the drainage of fluid out of the middle ear. If the drug/vehicle is very viscous and adhesive, it will resist drainage. In addition, the trabecular and concave anatomical structures near the oval and round windows can intensify this property. That is, a viscous vehicle that percolates through narrow slit-like spaces (between ossicles and the round window niche) will be resistant to draining away or evaporating. Gd in a vehicle that is adherent to the oval and round windows will have a much higher chance of diffusing into the SV and ST. As hMW HA and conventional HA have similar chemical compositions, the physical differences (molecular weight and viscosity) are the most rational explanation for the approximately two-fold higher enhancement with hMW HA.

The permeability of the round window is affected by many factors, including the size, configuration, liposolubility, and electrical charge of the drug, as well as the thickness of the round window membrane (26). The average thickness of the round window membrane is 70 μm in humans, while it is 12 μm in rats (27, 28). If the same drug is applied to the human and rat round windows, it will take a significantly longer time for the drug to permeate into the human inner ear compared to that of the rats. For instance, it took <1.0–1.5 min for the tracer (ferritin) to permeate into the inner ear in rodents. The same tracer was only occasionally found in the inner ear of primates after 1–3 h (29). In this study, it took about 2 h for the Gd to fully diffuse into the whole cochlea of a rat (when saline was the vehicle). The Nagoya group reported that the maximum inner ear enhancement can be achieved 24 h after intratympanic Gd injection (2). Our data are in line with previous studies that reported the difference of the round window permeability between humans and rodents. One of the main findings of our study is that the inner ear enhancement can be extended to ~7–10 days if hMW HA is used as the vehicle. This result is based on the permeability of the rat round window and may not directly apply to humans. Considering the slow diffusion of Gd through the human round window, the inner ear enhancement may last longer than 7–10 days in humans. But this point needs further verification by a clinical trial in the future.

In humans, the volume of the drug injected into the middle ear by intratympanic administration is usually 0.4–0.6 ml (30). In this study, the injected amount of hMW HA was 51.4 ± 10.9 μl, which is in line with a former publication: 50–200 μl in guinea pigs (31). We may presume that the 51.4 μL of hMW HA in rats can be converted to 0.4–0.6 ml in humans. That is, compared to rats, a 7.8–11.7 times larger dose of hMW HA may be needed to obtain a similar MRI image quality in humans.

Our results were consistent with previous studies also indicating that HA is an effective vehicle for Gd delivery, in that HA was able to improve the effects of Gd enhancement. It is assumed that HA can enhance the relaxivity of Gd and improve the signal-to-noise ratio during MRI (17, 32). When Gd was entrapped in 35-nm HA nanoparticles, a shorter relaxation time (better relaxation rate) was achieved with a 10-fold lower concentration of Gd (17). This will lead to improvements in the time window for clinical imaging acquisition due to the decrease in the scan time. In another study with a Gd-labeled peptide dendron–HA conjugate-based hybrid, the results showed that the longitudinal relaxivity increased three-fold (32). It also showed a two- to three-fold higher Gd accumulation in tumors, resulting in improved contrast enhancement and a much brighter SI on MRI (32). However, it should be noted that our results cannot be directly compared with these previous reports because we studied the effects of IT Gd delivery, whereas others examined the effects of Gd after systemic drug delivery (intravascular or intraperitoneal). We evaluated the cochlea as the target organ, whereas other reports presented details of *in vitro* or *in vivo* studies on tumors (e.g., breast cancer). In addition, Gd and HA were not bonded in this study (forming a mixture), whereas Gd was conjugated, entrapped, or dendronized with HA in the previous studies (forming compounds). The two- to three-fold greater enhancement effect may be similar, but the mechanism by which the enhancement occurred may be different between our study and previous reports.

Overall, hMW HA can be an effective vehicle for the inner ear delivery of Gd as well as other drugs. It would be highly advantageous to be able to deliver dexamethasone or methylprednisolone in a higher concentration and for a longer duration. Pharmacokinetic studies have shown that dexamethasone can be delivered into the inner ear for only several hours using standard-of-care IT methods (33). If the high efficiency of Gd delivery in this study is attributable to the physical properties of hMW HA, similar benefits may be obtained with other drugs loaded into hMW HA. Various drug delivery vehicles have been used to prolong the middle ear residence time and deliver a higher concentration of drugs into the inner ear. For example, gelatin (34), poloxamer 407 (31), collagen (35), methoxy polyethylene glycol-*b*-polycaprolactone block copolymer (11), and click-cross-linked HA (36) have been reported to have some positive effects. However, the clinical benefits in terms of hearing gain were not sufficient in most cases, reaching 10–25 dB at best (11, 37, 38). We surmise that hMW HA may be a useful candidate for overcoming this problem because the residence time of HA (24) is reported to be longer than those of chitosan glycerophosphate (39), poloxamer 407 (31, 40), and thiol-modified HA/gelatin hydrogel (41). Further studies are required to verify the treatment effects of using hMW HA as an IT vehicle for the delivery of therapeutic drugs such as dexamethasone and methylprednisolone.

The biocompatibility and safety of the vehicle should be assessed with additional considerations for IT drug delivery. The tympanic membrane is a thin layer (~100 μm thick) of cells that is very sensitive to infection and inflammation (42). If the vehicle is not completely biocompatible, it can cause adverse events such as acute otitis media, even if the same material does not cause significant complications in other parts of the body (11). For example, methoxy polyethylene glycol-*b*-polycaprolactone block copolymer is biocompatible when administered subcutaneously. However, a significant amount of inflammation was identified after IT administration of the same material (11). Thus, previous knowledge on the biocompatibility of various biomaterials may not apply in the ear, and each candidate material should be carefully reevaluated before being used as an IT vehicle. HA is highly biocompatible and has been approved by the US Food and Drug Administration for use in eye surgery and osteoarthritic pain (43). In the ear, we found that HA is one of the most biocompatible vehicles with no cases of inflammation, infection, or permanent perforation of the tympanic membrane (11). The excellent biocompatibility and high efficiency in drug delivery seem to make hMW HA an ideal IT drug delivery vehicle.

This study had some limitations. Firstly, the concentration of Gd was fixed. Gd enhancement can vary in diverse ways that are affected by both the vehicle and the concentration of Gd. We only tested one concentration of Gd because our goal was to verify the effects of the vehicles and not Gd. However, it would have been beneficial to further elucidate the interaction between the drug (Gd) concentration and the vehicle (hMW HA). Secondly, although MRI after IT Gd delivery is performed to determine the severity of EH in clinical cases, we were unable to evaluate this point because the animals in the present study had no pathological conditions in the ear. Once an optimal MRI protocol has been established (including the optimal vehicle, Gd concentration, and MRI time), we hope to quantitatively evaluate the cochlea of animals with EH. Thirdly, the results of our animal study may not be directly applicable to humans. The pharmacokinetics of Gd in the round window and the inner ear differ between humans and rats (4). The magnetic field strength (9.4 T in this animal study vs. 3.0 T in most clinical settings) of the MRI might have also affected the imaging outcomes. We assume that hMW HA would be a good vehicle for IT Gd delivery in humans, but this should be reevaluated before clinical use.

## Data Availability Statement

The raw data supporting the conclusions of this article will be made available by the authors, without undue reservation.

## Ethics Statement

The animal study was reviewed and approved by the Institutional Animal Care and Use Committee of Seoul National University Hospital.

## Author Contributions

Y-JH, MoP, MiP, JL, SO, and M-WS designed the study and analyzed the data. M-WS, MoP, JL, and SO supervised the experiments. Y-JH and M-WS wrote the main manuscript text. Y-JH and MiP performed the experiments. All authors contributed to the article and approved the submitted version.

## Conflict of Interest

Y-JH and M-WS hold a patent related to a hyaluronic acid-based drug delivery vehicle (Korean Intellectual Property application number 10-2020-0012749). The remaining authors declare that the research was conducted in the absence of any commercial or financial relationships that could be construed as a potential conflict of interest.

## Abbreviations

IT: intratympanic
Gd: gadolinium
EH: endolymphatic hydrops
MD: Ménière's disease
MRI: magnetic resonance imaging
ST: scala tympani
SV: scala vestibuli
hMW: high molecular weight
HA: hyaluronic acid
DTPA: diethylenetriamine pentaacetic acid
TR: repetition time
TE: echo time
FA: flip angle
FOV: field of view
TH: slice thickness
SM: scala media
SI: signal intensity.
